# Breast Radiotherapy-Related Cardiotoxicity. When, How, Why. Risk Prevention and Control Strategies

**DOI:** 10.3390/cancers13071712

**Published:** 2021-04-04

**Authors:** Ana Aurora Díaz-Gavela, Lourdes Figueiras-Graillet, Ángel Montero Luis, Juliana Salas Segura, Raquel Ciérvide, Elia del Cerro Peñalver, Felipe Couñago, Meritxell Arenas, Teresa López-Fernández

**Affiliations:** 1 Radiation Oncology, Hospital Universitario Quirónsalud Madrid, 28223 Madrid, Spain; elia.delcerro@quironsalud.es; 2 Radiation Oncology, Hospital La Luz, 28003 Madrid, Spain; 3 Clinical Department, Faculty of Biomedicine, Universidad Europea de Madrid, 28670 Madrid, Spain; 4 Cardiooncology Clinic, Centro Estatal de Cancerología Miguel Dorantes Mesa, Xalapa-Enríquez 91130, Mexico; figueirasgraillet.cardio@gmail.com; 5 Radiation Oncology Department, Hospital Universitario HM Sanchinarro, 28050 Madrid, Spain; amontero@hmhospitales.com (Á.M.L.); rciervide@hmhospitales.com (R.C.); 6 Cardio-oncology Unit, Hospital San Juan de Dios, San José 10103, Costa Rica; juliana.salassegura@ucr.ac.cr; 7 Cardiology Department, Hospital Clínica Bíblica. San José 10103, Costa Rica; 8 Radiation Oncology, Hospital Universitari Sant Joan de Reus, 43204 Reus, Spain; meritxell.arenas@urv.cat; 9 Universitat Rovira i Virgili. IISPV, 43204 Reus, Spain; 10 Cardio-oncology Unit. Cardiology Department, Hospital Universitario La Paz, 28046 Madrid, Spain; teresa.lopez@salud.madrid.org; 11 Hospital La Paz Institute for Health Research—IdiPAZ, 28046 Madrid, Spain

**Keywords:** breast cancer radiotherapy, cardio-oncology, radiation-induced heart disease, cardiovascular risk, prevention, cardiotoxicity, breast radiobiology, hypofractionated radiotherapy, coronary disease

## Abstract

**Simple Summary:**

Given that the majority of breast cancer patients receive adjuvant radiation therapy and/or systemic treatments that can enhance cardiovascular risk, it is imperative to consider a multidisciplinary approach to cardiovascular care and to develop strategies to identify and prevent radiation-associated cardiotoxicity. In this review, we seek to analyze the evidence of the mechanisms underlying cardiac damage secondary to breast irradiation, the impact of factors related to the patient herself, and the treatments administered, as well as to describe different strategies for reducing risks and managing radiation-induced heart disease if it has already occurred. All this with the aim of improving not only survival but also the quality of life for our patients.

**Abstract:**

In recent decades, improvements in breast cancer management have increased overall patient survival; however, many cancer therapies have been linked to an important risk of cardiovascular adverse events. Cardio-oncology has been proposed as an emerging specialty to coordinate preventive strategies that improve the cardiovascular health of oncologic patients. It employs the most suitable personalized multidisciplinary management approach for each patient to optimize their cardiovascular health and improve their survival and quality of life. Radiotherapy is an essential part of the therapeutic regimen in breast cancer patients but can also increase the risk of cardiovascular disease. Therefore, minimizing the negative impact of radiation therapy is an important challenge for radiotherapy oncologists and cardiologists specializing in this field. The aim of the present review is to update our knowledge about radiation-induced cardiotoxicity in breast cancer patients by undertaking a critical review of the relevant literature to determine risk prevention and control strategies currently available.

## 1. Introduction and Background

Breast cancer (BC) is the main cause of death in women in developed countries, and its incidence has been increasing even further in recent years [1]. BC management requires a multidisciplinary approach in which surgery, systemic treatments (chemotherapy, hormonal therapy and/or targeted therapies), and radiotherapy (RT) play well-established roles [2,3,4]. The positive impact of adjuvant RT, quantified in terms of local control and overall survival, has been demonstrated in randomized long-term clinical trials [5,6,7]. Since a high percentage of patients are long-term survivors, recent research has focused on describing and quantifying potential chronic adverse effects of RT on quality of life (QoL) and/or overall survival [8]. Cardio-oncology is a multidisciplinary field concerning the prevention, diagnosis, control, and management of cardiovascular dysfunction derived from cancer therapies. Its main goal is to optimize cancer treatments so that patients receive the best possible antineoplastic therapies, minimizing adverse events and/or treatment discontinuation. The aim of our review is to provide well-structured information about the mechanisms underlying radiation-induced injury in BC, the impact of factors intrinsic to patients and/or of other cancer treatments, and to describe different risk-control strategies from a multidisciplinary perspective.

### 1.1. Physiopathologic Aspects

**Figure 1:**
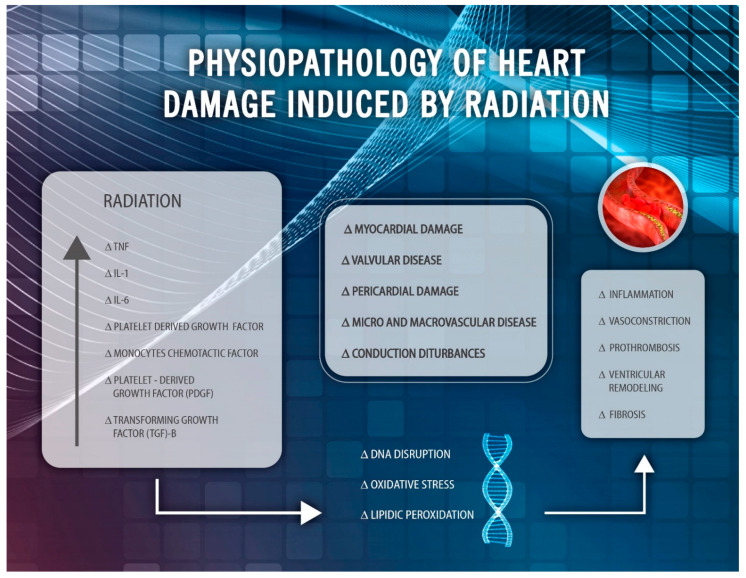
Physiopathology of radiation-induced heart disease (RIHD). IL: interleukin; TNF: tumor necrosis factor.

Radiation-induced heart disease (RIHD) was first described in the 70s. The underlying mechanism combines an acute inflammatory response of the irradiated tissue with an increased release of inflammatory cytokines and growth factors (GF), such as tumor necrosis factor (TNF), interleukin (IL)-1, IL-6, and platelet-derived growth factor (PDGF) [9]. Direct vascular endothelium damage favors platelet activation and the loss of endothelium-derived vasodilator factors, such as nitric oxide (NO). Vasoconstriction and prothrombosis lead to hypoperfusion, microvascular thrombosis, and ischemia-induced cell death. Vascular injury has been observed by reactive oxygen species (ROS) that produce disruption of the DNA and persistence of the inflammatory response, favoring secondary intimal hyperplasia and development of atherosclerotic plaque. Activation of the myofibroblasts causes an increased collagen synthesis in the extracellular matrix and subsequent deterioration of the elastic structure of the vessel, resulting in arterial rigidity [10]. The changes derived from inflammation and oxidative stress, associated with high cholesterol levels, can facilitate lipid peroxidation and the production of foam cells that trigger a process of accelerated atherosclerosis, with thickening and fibrosis of the tunica media and adventitia [11]. Parallel to this, myocardial tissue can be replaced by fibrotic tissue composed of bands of collagen, resulting in local alterations that can favor ischemia, ventricular remodeling, heart failure, and cardiac wall motion abnormalities [12]. Histopathological abnormalities include diffuse fibrosis in the myocardial interstitium, with narrowing of the vascular lumen. The mechanism of injury is complex and multifactorial, with inflammation playing a key role in triggering different pathways, which finally lead to cell and tissue injury (Figure 1). In summary, the development of RIHD is a slow but constantly progressing process as a result of the activation of acute inflammatory pathways, causing a chronic pathogenic cascade.

### 1.2. Clinical Evidence

Recent registries have shown that BC patients treated with RT have a greater risk of developing cardiovascular events compared with women with no breast cancer history and no prior RT [13]. Radiation involves several deleterious effects on the heart, from DNA disruption to symptomatic clinical disease. Although cell damage can be observed immediately after RT, cardiotoxicity manifests years after the exposure to ionizing radiation. It is difficult to establish the real incidence of RIHD as well as prospective risk scores because oncologic registries do not generally include patient comorbidities or real RT dosimetric data, especially in patients treated in the pre-three-dimensional-RT (3D-RT) era [14]. However, an anatomical advantage in BC patients provides us with interesting information from clinical trials and population registries alike. A more robust evaluation of RIHD can be performed by comparing right or left BC populations since cardiac radiation doses differ in the left breast (L-Br) versus right breast (R-Br) cancer [15,16]. Studies focusing on RIHD are summarized in Table 1.

McGale et al. concluded that overall survival was independent of laterality, but cardiac events were more frequent in the L-Br RT group, especially with pre-existing cardiovascular comorbidities (myocardial infarction (1.22 [95% CI 1.06–1.42]), angina (1.25 [1.05–1.49]), pericarditis (1.61 [1.06–2.43]) and valve diseases (1.54 [1.11–2.13])) [17]. Other groups reported myocardial perfusion defects [18], as well as cardiac wall motion abnormalities and impaired ventricular function in patients receiving L-Br-RT [19].

In 2013, Darby et al. established a link between the mean radiation dose received by the heart (mean heart dose: MHD) and the probability of suffering a cardiac event. The authors concluded that in patients treated with pre-3D-RT techniques, the relative risk of suffering an acute ischemic event was 7% for every 1 Gy increase in MHD, with no clear, safe threshold. The risk increase started within the first 5 years after RT and continued into the third decade after the treatment. [20]. Nonetheless, despite the high quality of this study, it presented some weaknesses. Due to its retrospective nature, baseline CV risk was not known, real dosimetric data were not available in all patients (MHD was estimated), and there was an imbalance of comorbidities among treatment groups. In 2017, Van der Bogaardt et al. validated Darby’s model in a cohort of patients treated with 3D-RT, for whom real dosimetric data were available. The authors concluded, after a nine-year follow-up, that the cumulative incidence of coronary events increased by 16.5% per Gy of MHD and that the volume of left ventricle receiving 5 Gy was the best predictor of risk [21].

In a recent meta-analysis, Cheng et al. concluded that patients receiving L-Br RT were at significantly higher risk of acute cardiovascular events within the first decade after exposure and for mortality from the second decade [22], and Taylor et al. and Henson et al. studied the impact of different cardiovascular risk factors that increased RT-specific mortality: tobacco and receiving chemotherapy treatment, respectively [23,24]. 

Sardar et al. evaluated the impact of patient follow-up time, concluding that patients treated before the 1980s and those with follow-up over 15 years were the patients with the highest risk of cardiovascular disease [25]. On the other hand, Wennstig et al. brought into the equation the influence of radiotherapy treatment volumes on cardiac risk, with a higher relative risk in those patients with significant lymph node involvement in whom locoregional lymph node areas had to be irradiated [26].

However, recent evidence suggests a reduced impact of contemporary RT on cardiovascular mortality [27]. Some of the latest published data come from the Danish Breast Cancer Group, which analyzed 22,056 patients receiving RT. While they found a trend toward an increased risk of coronary artery disease in left-sided versus right-sided BC in patients irradiated with non-computed tomography (CT)-based techniques or BC patients irradiated with CT-based techniques, no trend toward an increased risk of coronary artery disease in L-Br versus R-Br patients was observed within the first 10 years. Moreover, at a median follow-up of 8 years, the risk of valvular heart disease was not associated with the laterality of the irradiated breast [28].

For all the above reasons, it is essential to aim to increase the precision of RT delivery and to optimize strategies for risk evaluation, follow-up, rehabilitation, and global management of RIHD.

## 2. Baseline Risk Evaluation

The risk of RIHD is a dynamic variable and depends on prior and ongoing systemic cancer therapies and modifiable (lifestyle-dependent) and non-modifiable (sociodemographic, previous heart disease) risk factors (RF) [29]. Risk factors for RIHD are summarized in Table 2. Optimizing the management of cardiovascular risk factors (Table 3) and/or underlying heart disease may reduce the risk of cancer therapy-related cardiovascular events and the risk of early antitumoral treatment discontinuation [30,31,32,33].

## 3. Specific Risk of Cardiotoxicity in Breast Cancer Patients Receiving RT

**Figure 2:**
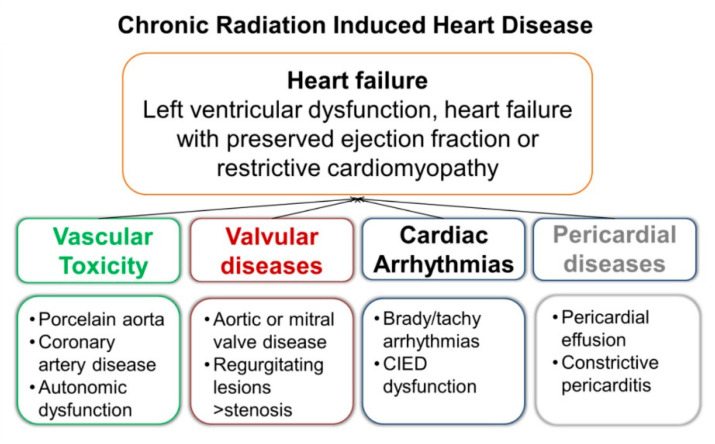
Chronic radiation-induced heart disease.

RIHD includes a wide range of cellular, metabolic, and structural complications in highly radiosensitive tissues. All cardiac structures are susceptible to radiation injury, which can be exacerbated by systemic treatments. RIHD includes valvular heart disease, premature and accelerated atherosclerosis, cardiac arrhythmias, autonomic dysfunction, pericardium diseases and heart failure. Irradiation can also damage cardiac implantable electronic devices. (Figure 2) [34,35].

### 3.1. Valvular Heart Disease

The prevalence of RT-induced valvular disease in BC ranges between 0.5 and 4.2% [36]. Lesions are characterized by thickening and accelerated calcification of the valves leading to stenotic or regurgitant defects. RT-associated valvular calcification may also involve adjacent structures such as the subvalvular apparatus and the annulus. The underlying mechanism involves fibroblast proliferation leading to increased production of osteogenic factors that induce valvular calcification.

The incidence of valve disease induced by breast RT does not reach levels reported in Hodgkin’s lymphoma patients as the radiation doses used for BC are lower than in thoracic or mediastinal irradiation (usually over 30 Gy) [37]. Valves on the left side are the most frequently affected, and calcification of the mitral-aortic junction is a hallmark of post-radiation injury. The incidence of valvular heart diseases rises significantly after 20 years, causing mild aortic stenosis in 45%, moderate in 15%, and severe in 15%. The incidence of mild mitral insufficiency can reach 48%. The risk of radiation-induced valvular heart disease is higher in left BC, patients treated with cobalt and 2D-RT techniques, doses >30–35 Gy, and patients of young age at the time of exposure, or when using concomitant cardiotoxic medication [10,38]. In patients with severe aortic stenosis, the porcelain aorta should be ruled out before valvular intervention, and percutaneous techniques should be considered in patients with an expected survival >12 months [9].

### 3.2. Coronary Artery Disease

The link between RT and coronary artery disease is well-established, and this is one of the main causes of cardiac mortality in patients with BC. In 1992, Rutqvist et al. found that the risk of death from ischemic heart disease was 3.2 times greater (*p* < 0.05) in women who had received L-Br-RT than in the group with no prior radiation [39]. In fact, a history of thoracic RT per se is a risk factor for the development of coronary artery disease. Women with BC who receive RT have a 30% higher risk of developing coronary disease and a 38% higher risk of cardiovascular death than non-irradiated women [26].

**Figure 3:**
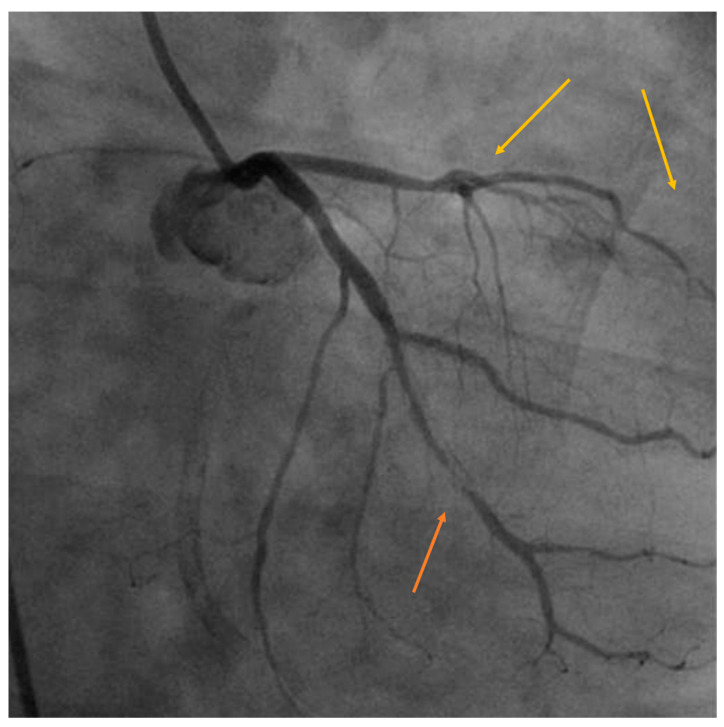
Coronary artery disease in a left breast cancer patient without cardiovascular risk factors treated with chemotherapy and radiotherapy. A long lesion with severe stenosis in the mid-distal portion of the circumflex artery (orange arrow). The left anterior descending artery (yellow arrows) also has a diffuse disease and severe stenosis (95%) in the mid-distal segment. Image courtesy of Dr. Andrés Daniele. “Ángel Roffo” Oncology Institute Buenos Aires, Argentina.

The endothelial cell is highly radiosensitive, and doses higher than 2 Gy can trigger inflammatory cascades that favor atherosclerosis. When combined with the prothrombotic condition of cancer, it can lead to conditions such as angina, acute coronary syndrome, myocardial infarction, malignant arrhythmias, and death. Coronary involvement is anatomically correlated with the site of radiation, and in left BC, lesions are most commonly found at the level of the left main coronary artery, the ostia, or the anterior descending artery in the mid and distal segments, the diagonal branches, and in the proximal segment of the right coronary artery. Atheromatous plaques tend to be longer, of tubular appearance, soft, with more fibrotic than lipidic contents, and frequently present intimal hyperplasia. Figure 3 graphically illustrates the late coronary disease in a 56-year-old patient without basal cardiovascular risk factors diagnosed with triple-negative breast cancer and treated with anthracyclines, paclitaxel, and RT (45 Gy) to the whole breast. It is complex to identify the ultimate cause of this patient’s coronary artery disease because RT has a synergistic effect with chemotherapy, which itself has a proven coronary deleterious effect, but what is striking, in this case, is the distribution of the lesions, both in length and location, matching precisely with the irradiated area. Although its therapeutic management is the same as for conventional treatment (in patients with no prior RT), revascularization should be evaluated in the presence of acute coronary syndrome or myocardial infarction [9,20,22]. Whereas the link between coronary artery disease and previous irradiation is well-established, the risk of death has diminished significantly over the years, and long-term follow-up of patients treated with contemporary RT techniques could provide some interesting answers.

### 3.3. Pericardiac Disease

Before the 1970s, post-RT acute pericarditis was quite common. Today, however, its incidence has decreased substantially due to lower radiation doses and the use of highly conformal techniques [9]. Even so, this entity should be included in the differential diagnosis of patients presenting chest pain, fever, pericardial rub, and electrocardiographic alterations in the weeks following treatment. This symptomatology can be caused by RT inflicting direct damage on the pericardium or by inflammation and necrosis of adjacent tissues. Chronic pericarditis consists of a thickening of the pericardium caused by the chronification of an inflammatory process. By contrast, constrictive pericarditis, diagnosed quite often in other thoracic tumors such as lung cancer or Hodgkin’s lymphoma (reaching 10–20% at >5 years after treatment for doses >50 Gy), is anecdotical in BC [11,12].

### 3.4. Conduction Disorders

The combination of RT and chemotherapy increases the risk of bradyarrhythmias. Although in BC patients this RT-associated risk is low, electrocardiographic monitoring is recommended, especially in patients with comorbidities or atherosclerotic disease. Atrial fibrillation is the commonest arrhythmia described after cancer treatments for BC. Varying degrees of atrioventricular block (especially complete block of the right branch owing to the anatomical proximity to the right ventricle), supraventricular arrhythmias, ventricular tachycardia, sick sinus syndrome, or QT prolongation have also been described after radiation and should be closely monitored [40]. The same applies to persistent sinusal tachycardia and loss of cardiovascular circadian rhythm, bearing in mind that persistent tachycardia can degenerate into tachycardiomyopathy [9,11,38]. In a series of nearly 200 breast cancer survivors, conduction abnormalities were found at 6 months and at 10 years after treatment. Nineteen percent of patients had pre-treatment conduction abnormalities, which increased to 45% at 6 months and 10 years after therapy. The predominant changes at 6 months were T wave abnormalities in left-sided breast cancer patients. At 10 months, there were fewer T wave changes but increased ST depression. Most of these changes were transient and had no clinical relevance [41].

### 3.5. Myocardial Injury

Heart failure with preserved ejection fraction appears to be more common than systolic dysfunction in patients with prior RT. Micro- and macrovascular involvement and direct damage to the cardiomyocyte tend to favor myocardial fibrosis and ischemia. Concomitant treatment with cardiotoxic drugs such as anthracyclines and trastuzumab potentiates damage to cardiac muscle. Myocardial perfusion defects have also been found in patients subjected to RT, suggesting damage to the microvasculature that could lead to diastolic dysfunction [42].

### 3.6. Implantable Cardiac Stimulator Device Dysfunction

Although acute toxicity from RT (pericarditis) in BC is rare, radiation can cause dysfunction of implantable cardiac stimulator devices (ICSD), which can be transitory, resetting to baseline, or can permanently damage the device [43]. The main factor associated with this effect appears to be the energy of the beam, being recommendable to use ≤6 MV and not surpass 2 Gy in pacemakers and 1 Gy in defibrillators (IAD) [44]. These devices should be checked regularly in patients both during RT and after its completion, especially if they are pacemaker-dependent or present cardiovascular symptomatology [45,46].

## 4. Toxicity Enhancement by Systemic Treatments

Systemic regimens used in BC treatment frequently combine pharmaceutical drugs such as anthracyclines (AC), cyclophosphamide, anti-human epidermal growth factor receptor 2 (Anti-HER2), fluoropyrimidines (5-FU, capecitabine), taxanes, antiangiogenics, and hormonal treatments, all known to have harmful effects on the myocardium [47,48,49].

Although an in-depth analysis of this is outside the scope of this study, it is pertinent to briefly summarize the pharmaceutical drugs most used and the mechanisms of injury to the cardiovascular system that can even affect patient survival (Table 4).

Anthracycline-induced cardiotoxicity seems to be associated with cardiac dysfunction in 2.2–10% of patients and has been categorized into acute (usually reversible decline in myocardial contractility), early-onset chronic progressive (congestive cardiomyopathy), and late-onset chronic progressive that eventually may lead to heart failure [50,51,52]. The use of the humanized monoclonal antibody trastuzumab has meant a paradigm shift in the management of HER2-positive breast cancer patients; some studies suggest that Anti-HER2 secondary cardiotoxicity (ranging from a decrease in the left ventricular ejection fraction to heart failure) occurs in approximately 10% of patients, manifests during the treatment, is not dose-dependent, increases significantly with the concomitant use of anthracyclines, and is usually reversible [53,54,55]. Cyclophosphamide cardiotoxicity is rare in breast cancer patients, but it has been associated with hemorrhagic and fulminant myocarditis, fibrinous pericarditis, and acute heart failure [56,57]. Bevacizumab is frequently prescribed as a first-line treatment in metastatic BC in combination with other agents. In high doses, it increases the risk of cardiovascular adverse events, both venous and arterial, particularly ischemia, bleeding, and hypertension [58]; it is also associated with left ventricular dysfunction in 2% of patients and NYHA III-IV clinical heart failure in up to 1% (BEATRICE study) [59]. The incidence of myocardial ischemia secondary to fluoropyrimidines varies depending on dose, scheduling, and route of administration; in addition, between 3–8% of the population are dihydropyrimidine dehydrogenase deficient (DPD) and potentially exposed to increased toxicity, although its impact on cardiotoxicity is still under study [60]. The use of taxanes is related to rhythm disturbances as asymptomatic sinus bradycardia, conduction blocks, and manifestations of cardiac ischemia, but cardiotoxicity is often resolved after the termination of the treatment [56]. Endocrine agents have a favorable toxicity profile compared to chemotherapy or targeted therapies. Tamoxifen is most frequently associated with higher thrombotic risk compared with aromatase inhibitors, while the latter are more frequently associated with grade 3–4 coronary events as well as alterations in the lipid profile [61,62].

## 5. Risk Reduction Strategies

The World Health Organization (WHO) defines three basic levels of disease prevention [63] that can be applied to manage toxicities resulting from cancer therapies. The first of these is to prevent the damage (primary prevention). Then, early detection favors the implementation of measures to alter its natural course (secondary prevention), and, finally, once the damage has been done, strategies must be aimed at reducing the impact of the sequelae (tertiary prevention). Focusing specifically on BC and RIHD, there is, in fact, little evidence for secondary prevention. Owing to a lack of specific validated biomarkers for RIHD and also of imaging techniques with sufficient sensitivity and specificity to detect subclinical disease, our options are limited when attempting an early diagnosis in asymptomatic women upon whom specific corrective measures could potentially be used [64]. In addition, we have no cardioprotective pharmacological treatments proven to be effective at preventing or altering the natural history of RT injury [65,66], and evidence of the impact of acting upon baseline cardiovascular risk factors is lacking. Moreover, the fact that this corresponds to a chronic adverse event with a latency of years and a low prevalence makes it difficult to study the impact of these interventions in clinical trials. Therefore, current prevention strategies for RIHD in BC consist mainly in performing highly conformed RT treatments with cardiac preservation techniques and investigating the potential role of altered radiotherapy fractionations [67].

### 5.1. Control of Cardiotoxicity Factors Associated with Radiotherapy Treatment

Parallel to the progress in systemic regimens, irradiation techniques in BC have also significantly improved, and risk estimations reported in classical studies cannot be extrapolated to current treatments. CT is one of the pillars of cancer therapy. The emergence of 3D-RT represented a radical change in practice, particularly for the radiation oncologist. It permitted target volumes and organs at risk to be defined in 3D by drawing contours on CT images on a slice-by-slice basis, as opposed to drawing beam portals on a simulator radiograph as they used to. During the planning process, it helps to identify not only the heart and its substructures but also the lungs, ribs, and other surrounding organs, with the assistance of numerous contouring atlases [68,69,70,71]. From the dosimetric point of view, high-dose regions are conformed much more closely to the target volume than was previously possible, thus reducing the volume of normal tissues receiving high doses. With the development of new therapeutic modalities, irradiation doses to adjacent healthy organs (organs at risk: OAR) can also be reduced [72], and new fractionation schemes offer radiobiological advantages in terms of the impact of RT.

#### 5.1.1. Maneuvers to Separate the Heart from the Chest Wall

Among the maneuvers that allow the chest wall to be separated from the heart are irradiation in the prone position as well as techniques for monitoring respiratory movement [73,74,75].

The rationale behind prone position irradiation is that in this position, the breast gland is separated further from the chest wall, thus potentially reducing the cardiac dose [76]. One of its main benefits is that there is less movement of the chest wall while breathing compared to the supine position. One study at New York University (NYU) on 100 patients in both the supine and prone positions found that 85% of patients with L-Br BC had a mean of 11 cc less cardiac volume inside the irradiation field [77]. Nonetheless, drawbacks to this technique include the difficulty of reproducing this position and the fact that it is uncomfortable for patients [78]. However, a study by Stegman et al., with a longer follow-up (a median of 4.9 years) in a cohort of 245 patients, showed similar clinical efficacy for both positions but a better tolerance of the prone than the supine position [79].

The two techniques used to monitor respiratory motion are deep inspiration breath hold (DIBH) with or without an active breathing coordinator (ABC) and respiratory gating. In both techniques, the heart and diaphragm move downward on breathing in, thus increasing the distance between the heart and the chest wall [80,81]. Many studies have demonstrated a significant decrease in MHD when using DIBH [82,83,84], so this technique should be used whenever possible.

#### 5.1.2. Conformal Radiation Therapy Techniques

While three-dimensional conformal radiotherapy (3DCRT) is applied in a robust fashion, usually with two opposed tangential fields and a uniform dose in each field, with intensity-modulated radiotherapy (IMRT), multiple photon beams are aimed from different directions and with adjusted intensities, allowing closer shaping to the target contour. IMRT uses a multileaf collimator (MLC) that shapes and creates non-uniform areas of radiation intensity in patients by adjusting the leaf speed [85]. The RT beam can therefore adapt to the anatomical curve, thus minimizing the dose received by adjacent healthy tissues. In spite of the adaptability of IMRT, conformal 3D-RT (the technique most widely used worldwide) can be optimized to a greater or lesser extent by using two simple parallel and opposing tangential fields until several subsegments are created from each one (field-in-field or also called FiF-IMRT), thus increasing the capacity to reduce the dose on the OAR. It is also important to distinguish between patients who only require adjuvant irradiation of the remaining breast and those who also need irradiation of the lymph node regions and even the internal mammary chain. In the latter, IMRT/volumetric modulated arc therapy (VMAT) would have the potential benefit of reducing the dose on the OAR in these patients [85]. In this regard, and although inverse planning (inverse-IMRT/VMAT/helical-tomotherapy) seems to provide a superior target coverage and heart sparing, many studies have addressed that, to achieve this, a larger volume of other OARs receive low radiation dose, especially lungs and the contralateral breast, which could lead to a higher incidence of secondary malignancies or unexpected late lung toxicity. Numerous studies have tried to clarify this question, but none have included enough patients to draw definitive conclusions. Based on this, and given that the most appropriate technique for left-sided breast cancer is still under debate, it is advisable to individualize the treatment and select, for each patient, the technique that provides us with the greatest logistical, clinical, and dosimetric advantages [86].

On the other hand, and using another type of particle, the advantage of proton therapy is in the intrinsic physical capacity of the protons to deposit their maximum dose inside the tumor (Bragg peak), with a rapid dose fall-off when reaching the OAR [87,88]. A lack of availability of this technique in most centers, in addition to its high cost, are major limitations for its widespread use.

#### 5.1.3. Reduction in Irradiated Volumes. Accelerated Partial Breast Irradiation. Intraoperative Radiotherapy

The partial breast irradiation (PBI) technique is based upon the knowledge that barely 3–4% of cases of local recurrence after conservative surgery occur outside the tumoral bed [89]. PBI consists of irradiating the surgical cavity with a safety margin [90]. Intraoperative radiotherapy (IORT) is perhaps one of the oldest forms of PBI and consists of administering RT on the tumorectomy bed during the surgical intervention, generally as a single fraction [91]. The rationale behind all these techniques is that by reducing the irradiation volumes, the dose received by adjacent healthy organs will be predictably lower.

### 5.2. Impact of Hypofractionated and Ultrahypofractionated Schemes on Cardiotoxicity by Radiotherapy

Hypofractionated (HFRT) and accelerated RT schemes can reduce the duration of RT from 25–28 sessions using the conventional fractionated treatment (CFRT) to just 15 sessions. Hypofractionation is now the standard of choice for adjuvant RT in BC, both after conservative surgery and also mastectomy [92]. The benefits of hypofractionated schemes are undeniable. A shorter total duration of treatments improves patients’ quality of life as well as reducing workloads and optimizing use of the radiation oncology services. Although initial reservations questioned the safety of these schemes that administer higher doses per fraction than those traditionally considered as acceptable, subsequent evidence appears to suggest the opposite.

The long-term results of START trials A and B demonstrated that a reduction in the number of fractions from 25 to 15, with a moderate dose increase from 2 Gy/day to 2.7 Gy/day [93], did not worsen breast cosmesis or increase the risk of complications in arm or shoulder [93]. Nonetheless, the potential risk of late-onset cardiotoxicity, especially in patients with left BC, has caused many oncologists to refrain from considering HFRT as the treatment of choice in daily practice. However, long-term studies with over 10 years of follow-up have not found evidence for an increased cardiac ischemic risk compared with CFRT [94]. In this line, the study of James et al., in New Zealand, which included 501 women treated between 2002 and 2006 with adjuvant HFRT (39–42.9 Gy/13–16 fractions) versus CFRT (50 Gy/25 fractions), found no differences in the incidence of ischemic heart disease, or any association with daily fraction size after a medium follow-up of >10 years [95]. The very thorough analysis of the Canadian studies found no evidence for increased cardiovascular risk factors (CVR) with HFRT either, and the Ontario Clinical Oncology Group observed no differences in death from cardiovascular causes after more than 10 years of follow-up [96]. Finally, a recently published meta-analysis found no differences in late-onset cardiotoxicity with postmastectomy RT, independently of the scheme used [97].

From a radiobiological perspective, there is no justification for ruling out using HFRT schemes because of a hypothetical rise in RIHD. In fact, radiobiological evidence seems to support its use. The linear quadratic model (LQ) is probably the most used formalism to calculate the radiotherapeutic isoeffective dose for different fractionation schemes and/or durations. The main parameters, α and β, represent the intrinsic sensitivity and have been defined both for different tumors and for normal healthy tissues. The α/β ratio for BC, estimated to be around 4 Gy or lower, favors a high tumor sensitivity at higher doses per fraction than conventional doses of 2 Gy/day and a reduction in total treatment time [98]. The concept of the biologically effective dose (BED) is based on this. This is the total dose required to produce the same logarithmic cell destruction between two schemes with different total doses, fractionations, and durations. A second concept called the equivalent total dose in 2 Gy fraction (EQD2) compares treatments administered with different doses and fractions with a conventional scheme of 2 Gy/fraction. The equations governing both concepts are:

BED = n × d(1 + d/α/β)

EQD2 = D × ([d + (α/β)]/[2 + (α/β)]);

where:

n = number of fractions,
D = total dose in Gy,
d = dose per fraction in Gy,
α/β = dose at which the linear and quadratic components responsible for cell death are equal. This indicates the intrinsic radiosensitivity of the tissue.

Late responding tissues such as healthy tissues and, in this specific case, cardiac structures, have low values of α/β. Generally, a value of 3 Gy is employed to estimate the dose delivered to healthy tissues and the potential risk of long-term toxicity. In an attempt to dissipate the fears of using HFRT schemes in BC, the group of Appelt et al. conducted an interesting study that compared the estimated fraction size-corrected doses to the heart with the four different HFRT schemes used in the START A and B trials and the Ontario Clinical Oncology Group versus CFRT. The authors demonstrated that the equivalent doses to the heart, assuming alpha/beta values between 3 and 1.5 Gy for healthy tissue, were lower with the HFRT schemes studied, showing them to be safer and to pose less potential risk [99].

Finally, HFRT in 15 fractions is starting to be replaced by irradiation schemes of the whole breast in five fractions, which will probably become the standard treatment in the near future. Published results already report on the safety of this technique. The randomized prospective U.K. FAST Trial, with three arms of 50 Gy in 25 fractions of 2 Gy versus five weekly fractions of 5.7 Gy or 6 Gy per fraction, included a total of 915 women with stage I or II BC after tumorectomy. With a mean follow-up of 10 years, the authors found no increased risk of ischemic heart disease toxicity with the ultrahypofractionated scheme [100]. The prospective randomized trial U.K.-FAST-FORWARD included more than 4000 women with invasive breast cancer (pT1-3pN0-1 M0) after surgery assigned to one of three arms: 40 Gy/15 sessions (control), 27 Gy/5 or 26 Gy/5 (1: 1: 1) to breast/chest wall, with a boost of 16 Gy/8 fractions or 10 Gy/5 fractions when indicated. With a median follow-up of 71.5 months, the authors identified no increased risk of RIHD or death from cardiac causes attributable to treatment with any of the schemes used [101].

Table 5 gives a clearer view of the differences in the BED and EQD2 values on the heart using moderate hypofractionation of 40.5 Gy/15 fractions or ultra-hypofractionation of 26 Gy/5 fractions of 5.2 Gy versus conventional schemes for an α/β value of 3 Gy, 2 Gy, and 1.5 Gy. The results show that equivalent doses to the healthy organ (heart) with HFRT are lower than with conventional schemes.

## 6. Potential Tools for Early Diagnosis, Monitoring, and Control of Post Breast Irradiation Heart Disease

As mentioned above, women with BC treated with RT are at higher risk of cardiac mortality than the general population. This risk depends upon the field and type of radiation, the mean heart dose administered, time since irradiation, and the presence of cardiovascular risk factors. The correlation between dose and cardiovascular events is linear, with no minimum safe dose [102].

**Figure 4:**
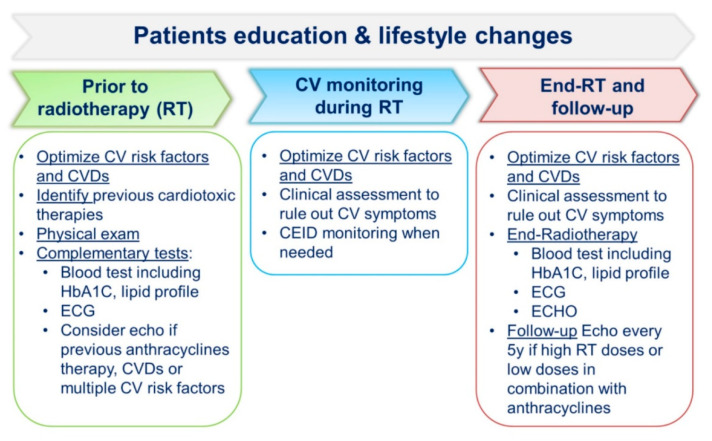
Radiotherapy treatment cardiac-monitoring strategies.

RIHD (Figure 2) usually appears several years after treatment, and the risk remains high for decades. For this reason, multidisciplinary strategies for prevention and early diagnosis are critical in BC patients (Figure 4). The baseline visit should establish an educational program, and all patients should receive structured advice on healthy lifestyle habits and treatment of cardiovascular risk factors [32]. Moreover, moderate-intense physical activity during the active treatment phase limits loss of functional capacity, improves treatment tolerance, and reduces depressive syndromes [56,103].

Treatment monitoring should be established in accordance with local resources and the patients’ vital prognosis [103]. It is essential to maintain a high level of clinical suspicion in patients with cardiovascular symptoms to avoid diagnostic delays. 3D-RT can be performed without evidence of early subclinical symptoms (such as changes in biomarkers or myocardial strain parameters), and there is no evidence for a need to monitor asymptomatic patients with imaging techniques during the active treatment phase [104].

It is essential to assess cardiovascular and functional status after RT to manage long-term monitoring [44,45,105]. Given that RT increases the vulnerability of the cardiovascular system, an annual check-up is recommended, including blood tests, ECG, and regular screening of ventricular function in survivors who have received radiation doses higher than 30 Gy or systemic treatment with AC. There is little evidence for the need to conduct periodic screening for ischemic heart disease in asymptomatic patients after treatment with RT [106]. Nonetheless, cardiac-CT is a very useful tool to detect early coronary artery disease in patients with angina symptomatology and before vascular surgery.

## 7. Cardiac Rehabilitation in Patients after Radiotherapy

Most patients with cancer experience physical and cognitive effects related to the treatment, which can have negative effects on their QoL and even reduce their overall survival. The chronic inflammation produced by cancer treatments involves metabolic, hypophyseal/hypothalamic effects promoting the release of oxygen free radicals [107]. Patients also experience a deconditioning in which unfavorable changes in body composition and muscle loss also cause fatigue and affect QoL [103]. Attempts have been made to objectify these effects using several parameters, including aerobic physical fitness, which decreases by amounts ranging from 5 to 26%, depending on the treatment regimens, and in which the inclusion of RT exacerbates these effects still further [107].

Furthermore, the effect on the patient’s physical condition is not limited to the time he/she is receiving active treatment. A study conducted by St. Jude Children’s Research Hospital in cancer survivors, including more than 1041 patients who had received AC or thoracic RT more than 10 years previously compared with control patients, demonstrated an important intolerance to exercise, defined as a maximum VO2 below 85%, associated with higher overall mortality of any kind [108]. Moreover, the CVR for this population group is higher, not only due to the presence of traditional risk factors but also because RT is associated with the development of coronary disease [109]. The positive effects of exercise have been reported in practically all types of cancer, not only because of an improvement in aerobic functional capacity but also owing to beneficial effects on other parameters such as blood pressure and lipids, all involved in the development of cardiovascular disease. The benefits include a greater mobilization of the white cell count, thus improving immunity, reduced expression of the CCAAT binding proteins (CEPBA) (usually increased during treatment), leading to increased proliferation of myocytes that reduces the toxic effects of the medications. In addition to improving cardiac contractility and preventing calcium overload, it also improves control of sarcoplasmic reticulum calcium cycling, ultimately protecting the heart and protecting against heart disease [110]. Similarly, an increased expression of antioxidant enzymes has been described, increasing the transcription of substances such as PGC-1 (peroxisome proliferator-activated receptor γ co-activator 1α), and a greater expression of vascular endothelial growth factor (VEGF), nitric oxide, and IL-6 [110].

We also know that preconditioning, or prehabilitation, is effective against the cardiotoxic effects of different antitumoral agents, so exercise should coincide with the start of treatment. Although no studies have specifically focused on the impact of rehabilitation in patients who have received RT for BC as the unique treatment as part of the therapeutic regimen, these patients could potentially benefit from targeted rehabilitation and physical training [111]. Patients must be assessed on a case-by-case basis, programming rehabilitation to suit each situation. In general, this therapy will consist of moderate aerobic exercise combined with resistance training and flexibility exercises before, during, and after the treatments. Inspiratory muscle exercises can also be included to improve functional capacity and VO2. [110,112]. All of this can help prevent cardiotoxicity, improve quality of life, and prevent future CV events.

## 8. Future Directions

As previously described, preclinical and clinical studies have identified different manifestations of RIHD in BC. However, in-depth knowledge of the biological mechanisms involved in RIHD is needed [113] to develop new non-invasive detection methods for diagnosis and follow-up. We need to identify specific biomarkers of RT-induced injury [64], to implement high-resolution imaging techniques in daily practice [114,115,116], and to combine the use of omics data with molecular imaging (single-photon emission computed tomography (SPECT-CT)) or positron emission tomography (PET-CT) for early RHID diagnosis [117,118].

It is essential to determine the precise radiation dose received by each cardiac substructure and its potential cardiovascular toxicity risk [119,120]. It is, therefore, recommendable to incorporate new consensual contour atlases into routine clinical practice, for which self-segmentation computer tools may be of help, to ensure the homogeneity of the dosimetric data compiled [121]. Clinical trials are also required to identify differences in radiobiological responses of cardiovascular tissues to different particles (photons versus protons/others) and their possible clinical impact [122].

The relative frequency of RIHD in patients with BC is low and manifests in the long-term. This implies that prospective trials would require a large number of patients and years of follow-up to reach sufficient statistical power and to obtain meaningful results about the interventions carried out. The design and monitoring of these is complex. If we conduct an active search for ongoing clinical trials (ClinicalTrials.gov) entering the following keywords in the search: breast cancer, radiotherapy, and cardiac toxicity, a total of 49 results are obtained; RIHD in BC is the main study object in only eight of these (Table 6). Because of these limitations, when attempting to construct quality evidence, it becomes even more necessary for all agents involved (cardiologists, radiation oncologists, and preclinical investigators) to adopt a proactive approach to emphasize the importance of research and investment in this field.

## 9. Conclusions

Although RT plays an undeniable role in BC survival, older studies conducted with obsolete non-cardioprotective RT techniques have demonstrated an increased cardiovascular risk after BC-RT without a safe radiation dose threshold, especially in patients with pre-existing comorbidities or those who receive concomitant or sequential systemic treatments. Fortunately, current technological and clinical advances allow for reducing RIHD risk by customizing the treatment, not only to each anatomical feature but also to each clinical risk, and cardio-oncology has been proposed as a new strategy to help prevent cardiovascular adverse events. We must bear in mind that the first cause of mortality associated with cancer is cancer itself, and the multidisciplinary collaboration between professionals, as well as patients’ education in a healthy lifestyle, plays an essential role in minimizing the potential morbidity and mortality derived from the treatments.

## Figures and Tables

**Table 1:** Summary of publications focusing on radiation-induced heart toxicity in breast cancer: ACE: acute coronary event; ChT: chemotherapy; CT: computed tomography; CV: cardiovascular; CVRF: cardiovascular risk factors; IHD: Ischemic heart disease; IR: incidence ratio; LV-V5: volume of the left ventricle receiving 5 Gy; MCE: major coronary events (myocardial infarction, coronary revascularization, or death from ischemic heart disease); MHD: mean heart dose; RR: relative risk; RS: risk score; RT: radiotherapy; 3D-RT: tridimensional radiotherapy; 2D-RT: two-dimensional radiotherapy; ys: years.

Study	Type of Study	Decade of RT/ RT Modality/ Dosimetry (RT Planning Data)	N. of Patients	Issue Evaluated	Effect
McGale 2011 [17]	Prospective. Population-based study	1976–2006 Various Estimated planning data	72,134 (34,825 received RT)	Incidence of heart disease: RT vs. no RT Left vs. right RT	IR: 1.08 for left vs. right RT For acute myocardial infarction, the increase in the IR, left vs. right, was greatest at 15+ ys after RT; for angina, it was greatest at 0–4 ys; and for pericarditis and valvular heart disease, it was greatest at 5–9 ys. Significantly higher IR of CV disease if RT was before 1990
Darby 2013 [20]	Retrospective. Population-based case-control study	1958–2006 Not reported Estimated planning data	963 (cases) vs. 1208 (controls)	MCE	Per 1 Gy of MHD: Increase of RR for MCE: 7.4%/16.3% Higher risk in the left breast and patients with CVRF
Sardar 2016 [25]	Metanalysis	?–2015 Various Not collected	289,109	CV mortality after left vs. right-breast RT	RR of CV mortality: 1.12 for left versus right RT RR of CV mortality: 1.23 after 15 years of follow-up Higher RR if treatment before 1982 (RR 1.38)
van den Bogaard 2017 [21]	Cohort. Single institution	2005–2008 3D-RT Individual planning data	910	Cumulative incidence of ACEs within 9 years of follow-up.	Per 1Gy of MHD: 16.5% increase for ACEs LV-V5: 29.3% vs. 16.9%: ACE vs. no event Developed an RS for predicting ACE, including age, CVRF, and LV-V5
Taylor 2017 [24]	Metanalysis	1972–1989 2D-RT Depends on the study	40,781	CV mortality Effect of smoking habit	RR cardiac mortality: 1.3 RT vs. no RT ↑ 4% mortality RR for each Gy of MHD Higher risk in smokers
Cheng 2017 [22]	Metanalysis	1980–2015 Various Not collected	1,191,371	CV disease CV mortality	RR CV mortality: 1.22 left vs. right RT RR CV mortality: 1.38 RT versus no RT IHD: RR 1.29 left versus right RT
Henson 2020 [23]	Registry-based Cohort	1987–2002 Various Not collected	1,018,505	Cardiac mortality	The RT- RR for cardiac mortality was greater at younger ages, lasted over 25 years, and was greater in women when ChT was also given
Wennstig 2020 [26]	Retrospective. Population-based case-control	1992 to 2012 3D-RT Not collected	361,008 (60,217 with BC + 300,791 without BC) Received RT: 37427	Risk of IHD	↑ risk left vs. right RT (HR 1.18; HRs increased with more extensive lymph node involvement and with the addition of systemic therapy) The cumulative IHD incidence started from the first years after RT and was sustained with longer follow-up
Louise Holm Milo 2021 [28]	Retrospective Population-based cohort	1999–2007: Non-CT-based 2008–2016: CT-based RT	29662 (22056 received RT)	Risk of MCE in left vs. right-sided BC patients treated during a non-CT vs. a CT-based period.	Non-CT period-> 15-year risk-difference left vs. right: *p* = 0.06. CT-based-> 10-year risk-difference left vs. right: *p*= 0.56

**Table 2:** Risk factors for radiation-induced cardiovascular complications BMI: body mass index; 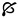: systolic blood pressure >140 mmg Hg and/or diastolic blood pressure >90 mm Hg, or on treatment; ✦: HbA1c > 7.0% or on treatment; Ø: LDL cholesterol level > 130 mg/dL or on treatment.

Risk Factors for Radiation-Induced Cardiovascular Complications
Lifestyle risk factors
Current smoker
Sedentary habit
Obesity (BMI > 30)
Demographic and CV risk factors
Age ≥65 years
Hypertension 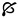
Diabetes mellitus ✦
Hyperlipidaemia Ø
Previous cardiovascular disease
Heart failure or cardiomyopathy
Coronary artery disease
Cardiac implantable electronic devices
Previous cardiotoxic cancer treatment
Prior anthracycline exposure (>250 mg/m^2^)
Prior thoracic radiotherapy

**Table 3:** Control objectives of cardiovascular risk factors in patients with cancer (adapted from ref. [30]). * The SCORE risk estimation system (the SCORE project) offers estimation of the risk of cardiovascular mortality at 10 years suited to the constraints of clinical practice and is calculated based on age, sex, smoking, systolic blood pressure, and cholesterol [33].

Cardiovascular Risk Factor	Treatment Goals	
Blood Pressure	<130/80 mmHg	
LDL Cholesterol	Very high risk or SCORE * ≥10%	<55 mg/dl
	High risk (SCORE * ≥ 5 y < 10%)	<70 mg/dl
	Moderate risk (SCORE * ≥ 1 y < 5%)	<100 mg/dl
	Low risk (SCORE * < 1%)	<116 mg/dl
Diabetes Mellitus	HbA1c < 7%	
Smoking	No	
Alcohol intake	<20 g/day in men and 10 g/day in women	
Exercise	Moderate physical activity at least 30 min/5 days a week	
Diet	Healthy diet	
BMI	20–25 kg/m^2^	

**Table 4:** Breast cancer systemic treatments that enhance cardiotoxicity: AI: aromatase inhibitors; Arrhyth: arrhythmia; ChT: chemotherapy; C-RF: associated risk factors that enhance cardiotoxicity; DPD: dihydropyrimidine dehydrogenase; Myo: myocarditis; Myo-C: cardiomiopathy; Myo-Is: myocardial ischemia; PDis: pericardial disease; T-DM1: trastuzumab emtansine; VE: vascular events; VEGF: vascular endothelial growth factor; π: cumulative % of patients with heart failure treated with doxorubicin according to the cumulative dose.

Cancer Treatment	Drug	VE	Myo-Is	Arryth	PDis	Myo	C-Myo	Mechanism	C-RF		
Anthracycline	Doxorubicin, Epirubicin,			Yes	Yes		Yes	Oxidative stress Alteration of DNA topoisomerase	Cumulative dose → dependent π	Dose(mg/m^2^)	%
										150	0.2
										300	1.7
										400	4.7
										500	15.7
										700	48
									Pre-existing heart disease High blood pressure Concomitant or sequential RT Age		
Anti-HER-2	Trastuzumab Pertuzumab, T-DM1						Yes	Disruption of the signal between the HER2 receptor and the neuregulin ligand.	27% of cardiac disfunction using Anti-HER2 + anthracyclines + cyclophosphamide concomitantly		
Alkilant agents	Cyclophosphamide	Yes	Yes	Yes	Yes		Yes	DNA alkylation.	Dose-dependant (>140 mg/kg) Advanced age Bolus administration Previous RT Concomitant ChT		
Fluoropirimidins (antimetabolites)	Capecitabine 5-FU		Yes			Yes	Yes	Binds irreversibly to the enzyme thymidylate synthase → inhibits cell division. Endothelial injury and thrombosis Increased oxygen consumption, oxidative stress, and vasospasm favored by the release of histamine	Continuous infusion Role of deficiency in DPD?		
Taxanes	Docetaxel Paclitaxel			Yes			Yes	By acting on microtubules decrease the concentration of calcium in cardiomyocytes, reducing the time from maximum contraction to relaxation	Use of AC concomitantly		
Endocrine agents	Tamoxifen	Higher thrombotic risk compared with AI						Block estrogen receptors in breast tissue	Baseline cardiovascular risk factors		
	AI	Slight but higher risk of acute myocardial infarction and heart failure compared with tamoxifen. Alterations in the lipid profile						Block the enzyme aromatase			
VEGF inhibitors	Bevacizumab	Yes	Yes				Yes	Bevacizumab binds to VEGF, inhibiting its ability to bind to and activate VEGF receptors → inhibition of angiogenesis	High-dose bevacizumab		

**Table 5:** Biologically effective dose (BED) and equivalent total dose in 2 Gy fraction (EQD2) differences between different fractionation schemes.

α/β	Variations	50 Gy (2 Gy/Fraction)	40.5 Gy (2.7 Gy/Fraction)	26 Gy (5.2 Gy/Fraction)
3 Gy	EQD2	50 Gy	46.2 Gy	42.6 Gy
	BED	83.3 Gy	77 Gy	71.1 Gy
2 Gy	EQD2	50 Gy	47.6 Gy	46.8 Gy
	BED	100 Gy	95.2 Gy	93.6 Gy
1.5 Gy	EQD2	50 Gy	48.6 Gy	49.8 Gy
	BED	116.7 Gy	113.4 Gy	116.1 Gy

**Table 6:** Ongoing trials in which radiotherapy and secondary heart damage are the main object of study. Source: ClinicalTrials.org: ACE: acute coronary event; BC: breast cancer; ChT: chemotherapy; CVD: cardiovascular disease; IBio: imaging biomarkers; GDF-15: growth differentiation factor-15; LVEF: left ventricular ejection fraction; MMPF: myocardial mitochondrial pyruvate flux; MP: myocardial perfusion; NTCP: normal tissue complication probability; NTproBNP: N-terminal pro B-type natriuretic peptide; PET-MRI: positron emission tomography-magnetic resonance imaging; PlGF: placental growth factor; PSLS: peak systolic longitudinal strain; RT-CVD: radiation-induced cardiovascular disease; RV- FAC: right ventricular fractional area change; SBio: serum biomarkers; ys: years.

Study/ Estimated Completion Date	Official Title	Type of Study	N. of Patients	Insight	Primary Outcome Measures	Secondary Outcome Measures
NCT03211442 Nov 2022	Implications of MEDIcal Low Dose RADiation Exposure - BReast Cancer Acute Coronary Events (MEDIRAD-BRACE): A Retrospective Cohort Study	Retrospective Cohort Study	7000	Externally validate multivariable NTCP models to assess the risk of ACE based on cardiac dose metrics in the first 10 ys after RT	ACE 10 ys after RT	Other cardiac complications RT-induced late non-cardiac toxicity
NCT02541435 Dec 2036	Acute and Long-term Cardiovascular Toxicity After Modern Radiotherapy for Breast Cancer —a Prospective Longitudinal Study	Observational. Cohort. Prospective	1600	Two cohorts of BC patients will be followed for the development of CVD for 15 ys	Incidence of CVD compared with corresponding estimates from the female general population 8 and 15 ys after RT	
NCT03748030 Dec 2021	Assessing Acute Cardiac Inflammation After Left-Sided Breast Cancer Radiotherapy With Hybrid PET/MRI	Observational. Cohort. Prospective	15	Identify the presence of acute low-dose RT-CVD in left-sided BC patients using hybrid PET/MRI	- Detection of IBio of acute/late cardiac inflammation: (FDG)-PET - Detection of IBio of acute/late cardiac perfusion changes: N-13 Ammonia PET - Detection of cardiac fibrosis: Gadolinium Enhanced MRI	
NCT02156648 Jul 2020	A Feasibility Study for Women Receiving Adjuvant or Neo-adjuvant Anthracycline Chemotherapy With or Without Radiation for HER2-neu Positive Invasive Ductal Carcinoma	Interventional (Clinical Trial). Single Group Assignment	20	To assess the feasibility of collecting plasma samples for SBio and to identify if there is an association between the SBio, echocardiography, and cardiac PET results in irradiated patients	Recruitment rates	Cardiotoxicity. To measure SBio, MP, RT-CVD, and PSLS
NCT04044872 Dec 2023	Single Institution Feasibility Study to Detect Radiation-Induced Cardiotoxicity in Receiving Thoracic Radiation Patients Using Hyperpolarized Carbon 13-Based Magnetic Resonance Spectroscopic Imaging	Interventional (Clinical Trial). Single Group Assignment	10	The goal of this study is to detect early changes in the mitochondrial metabolism in situ as a marker for subclinical RT-CVD	To determine if RT-CVD can be measured by an increase in [1–13 C]lactate/[13 C]bicarbonate ratio and a decrease in [5–13 C]glutamate formation	Determination of the prognostic value of decreased MMPF in predicting clinically significant RT-CVD
NCT03301389 Jan 2025	Cardiac Magnetic Resonance for Early Detection of Chemotherapy or Radiation Therapy Induced Cardiotoxicity in Breast Cancer (CareBest)	Observational. Cohort. Prospective	2000	Aimed to achieve early detection of CT or RT-CVD using T1 mapping MRI. To determine a prognostic imaging factor for treatment cardiotoxicity	Decrease in left ventricular ejection fraction(LVEF) 1 and 2 ys after RT	Major adverse cardiac events (MACE) 1 and 2 ys after RT
NCT04361240 Aug 2023	Cardiotoxicity in Breast Cancer Patients Treated with Proton or Photon Radiotherapy: A RadComp Ancillary Cohort Study	Observational. Cohort. Prospective	155	The investigators will collect SBio and echocardiograms prior to, during, and for up to 1 year following radiation for a subset of patients enrolled in RadComp study (NCT02603341)	LVEF RV-FAC Circulating NTproBNP Circulating PIGF Circulating GDF-15	Changes in: - LV systolic strain - Echocardiography derived Ventricular Arterial Coupling Measurement - Diastolic function (E/e’) - Circulating Troponin T(TnT) - Circulating high-sensitivity C-Reactive Protein (hsCRP)
NCT01758445 Jan 2030	Phase II Study of Postoperative, Cardiac-Sparing Proton Radiotherapy for Patients With Stage II/III, Loco-Regional, Non-Metastatic Breast Cancer Requiring Whole Breast or Chest Wall Irradiation With Lymph Node Irradiation	Interventional (Clinical Trial). Single Group Assignment	220	The study goal is to demonstrate a “meaningful benefit” of proton therapy for women with loco-regionally advanced BC	5-y determination of the rates of acute and late RT toxicities	To determine dose distribution of proton therapy to coronary arteries and heart. Determine the incidence of ACE, cardiac morbidity, and mortality
